# Selection for Silage Yield and Composition Did Not Affect Genomic Diversity Within the Wisconsin Quality Synthetic Maize Population

**DOI:** 10.1534/g3.114.015263

**Published:** 2015-02-02

**Authors:** Aaron J. Lorenz, Timothy M. Beissinger, Renato Rodrigues Silva, Natalia de Leon

**Affiliations:** * Department of Agronomy and Horticulture, University of Nebraska, Lincoln, Nebraska 68583; † Department of Plant Sciences, University of California, Davis, California 95616; ‡ Department of Agronomy, University of Wisconsin, Madison, Wisconsin 53706

**Keywords:** genomic diversity, plant breeding, silage composition, association mapping

## Abstract

Maize silage is forage of high quality and yield, and represents the second most important use of maize in the United States. The Wisconsin Quality Synthetic (WQS) maize population has undergone five cycles of recurrent selection for silage yield and composition, resulting in a genetically improved population. The application of high-density molecular markers allows breeders and geneticists to identify important loci through association analysis and selection mapping, as well as to monitor changes in the distribution of genetic diversity across the genome. The objectives of this study were to identify loci controlling variation for maize silage traits through association analysis and the assessment of selection signatures and to describe changes in the genomic distribution of gene diversity through selection and genetic drift in the WQS recurrent selection program. We failed to find any significant marker-trait associations using the historical phenotypic data from WQS breeding trials combined with 17,719 high-quality, informative single nucleotide polymorphisms. Likewise, no strong genomic signatures were left by selection on silage yield and quality in the WQS despite genetic gain for these traits. These results could be due to the genetic complexity underlying these traits, or the role of selection on standing genetic variation. Variation in loss of diversity through drift was observed across the genome. Some large regions experienced much greater loss in diversity than what is expected, suggesting limited recombination combined with small populations in recurrent selection programs could easily lead to fixation of large swaths of the genome.

Silage production is the second most important use of maize in the United States following grain production (USDA National Agricultural Statistics Service 2014). With the expected increase in consumption of animal, especially dairy, products worldwide, as well as regulations currently in place in the United States and other countries in the world related to the need to increase the presence of biomass-derived biofuel production, improving forage yield and composition has become an area of substantial research and development.

Maize silage is forage of high quality and yield (Coors and Lauer 2001). A major difference between maize silage and other types of forage relates to the contribution of the grain, which represents approximately 50% of the total biomass in average temperate maize hybrids in the United States. (Lorenz *et al.* 2010). Cell wall–bound carbohydrates provide another important energy source for ruminant animals. Breeding for silage production in maize, therefore, involves the simultaneous improvement of forage yield and cell wall composition. A substantial amount of work has been dedicated to establishing associations between the relative contribution of compositional properties—digestibility, carbohydrate concentration, and protein—to animal productivity (Schwab *et al.* 2003). Summative equations, such as MILK2006 (Shaver *et al.* 2006), combine forage composition and yield to calculate expected milk per hectare, which can be directly used as a selection criterion.

The Wisconsin Quality Synthetic (WQS) maize population was developed by the University of Wisconsin maize breeding program nearly three decades ago and is currently in its fifth cycle of recurrent selection for high-quality stover and high forage yield (Frey *et al.* 2004; Gustafson *et al.* 2010). Gustafson *et al.* (2010) evaluated forage yield and composition for each cycle of WQS *per se* as well as topcrosses to two commercial testers. Linear improvements were observed in whole plant yield, stover yield, and whole-plant composition both in the population *per se* as well as topcross evaluations. Although stover quality *per se* did not improve through selection, milk yield on the basis of Mg ha^−1^ has increased 24%. Changes in silage yield have been greater than changes in silage composition, suggesting that the current selection protocol tends to emphasize improvements in forage yield compared with composition (Gustafson *et al.* 2010). Eight inbred lines have been released from the different cycles of this population (W601S, W602S, W603S, W604S, W611S, W612S, W613S, and W614S) and made available to the public.

Recurrent selection in plant breeding is a cyclical process of evaluation, selection, and recombination practiced within a closed population with the goal of improving the mean population performance while maintaining genetic variation (Bernardo 2010). Maintaining genetic variation in a population undergoing recurrent selection is critical for continued response to selection, but achieving an intensity of selection sufficient for making genetic gain can be antagonistic to this goal. Studies reporting changes in average diversity at the molecular marker level within maize recurrent selection programs have been frequently performed and have found decreases in diversity in proportion to that expected through genetic drift alone (Butruille *et al.* 2004; Romay *et al.* 2012; Labate *et al.* 1999). Low marker densities prevented these studies from examining the distribution of the genetic diversity across the genome. This is important to examine because diversity in some genomic regions may have been maintained by chance, whereas diversity in other regions may have been completely lost by chance through the fixation of large swaths of the genome due to infrequent recombination. Only two to three crossovers per chromosome are expected in maize (Anderson *et al.* 2003)

Dense genotyping of populations undergoing recurrent selection can also be used for identifying signatures of selection, as has been performed with model organisms (Parts *et al.* 2011; Turner *et al.* 2011) and agricultural species such as maize (Wright *et al.* 2005; Hufford *et al.* 2012; Wisser *et al.* 2008; Coque and Gallais 2006; Falke *et al.* 2007; Hirsch *et al.* 2014; Beissinger *et al.* 2014). When combined with genomic information, an array of statistical methods, both widely recognized and recently proposed, hold great promise for identifying genes underlying phenotypic response to selection and impacts of selection on genomic structure (Lewontin and Krakauer 1973; Barrett and Hoekstra 2011). A disadvantage of selection mapping stems from the fact that selection is often not performed for a single trait, making it impossible to estimate effects of individual loci on specific traits.

Association mapping is another option for identifying loci underlying variation for traits of interest within breeding populations. A major setback of this approach, however, is low power to detect rare alleles for populations of moderate size (Myles *et al.* 2009). Another issue highly relevant to the application of association mapping to populations undergoing recurrent selection is the fact that alleles conferring favorable values for traits are expected to change in frequency through selection and thus contribute to structure between the different cycles of selection. When population structure is corrected for using a mixed linear model (Yu *et al.* 2006), power to detect these alleles contributing to genetic differences between cycles is reduced (Rincent *et al.* 2014). Wisser *et al.* (2011) proposed combining selection mapping and association mapping to overcome deficiencies of both methods for dissecting the genetic architecture underlying response to selection.

Assessing the impact of recurrent selection on the distribution of diversity across the genome would further the understanding of how drift and selection shape genomic architecture. Moreover, identifying genomic regions influencing forage composition and yield would be beneficial to silage breeding. With this in mind, the objectives of this study were to identify loci controlling variation for maize silage traits through association analysis and the assessment of selection signatures and to describe changes in the genomic distribution of gene diversity through selection and genetic drift in the WQS recurrent selection program. To accomplish this, individuals from multiple cycles of the WQS recurrent selection program were genotyped using a high-density SNP array. Phenotypic data were collated from historical records of the long-term WQS recurrent selection program.

## Materials and Methods

### Germplasm

**Figure 1:**
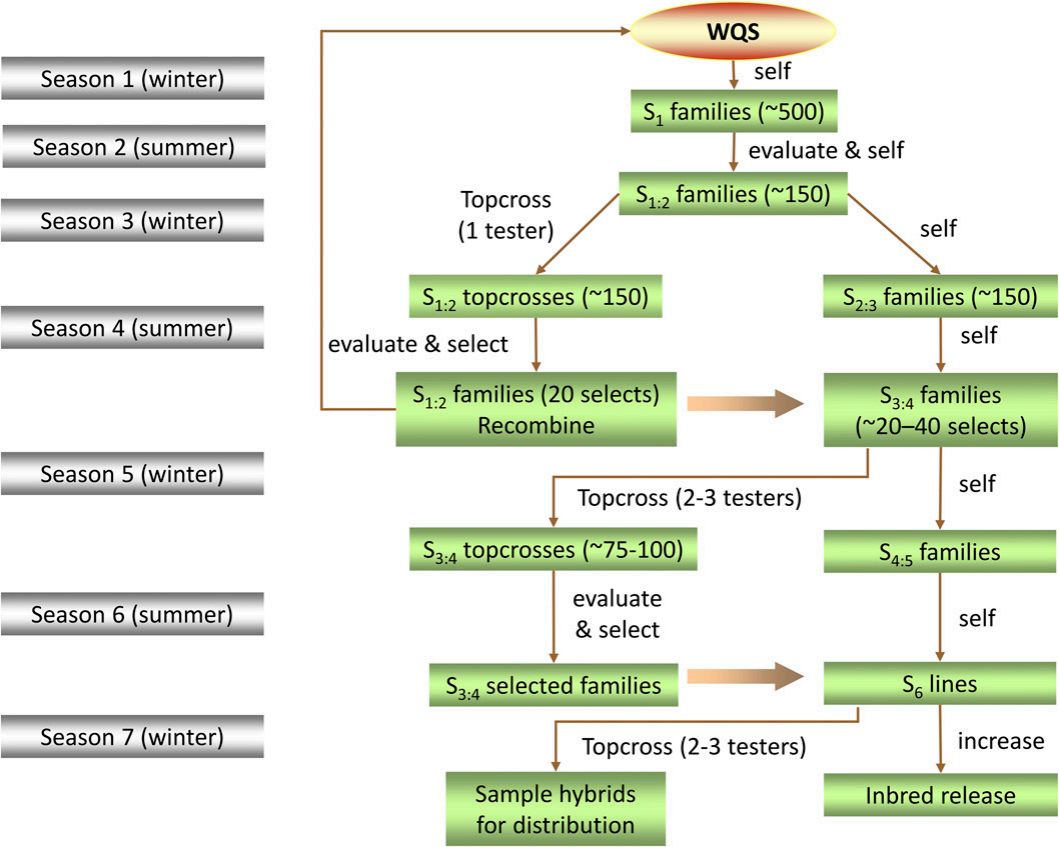
Schematic of the selection protocol utilized to advance the Wisconsin Quality Synthetic (WQS) population. A second generation (S_2_) topcross selection method is utilized to improve this germplasm. Inbreds derived from succeeding cycles of improvement are developed and released. Population improvement and inbred development occur simultaneously. The red oval highlights the approximately the 20 S_2_ families that originate each subsequent cycle of selection.

Details on the formation of WQS can be found in Frey *et al.* (2004) and Gustafson *et al.* (2010). The breeding protocol utilized to advance WQS is depicted in Figure 1. Briefly, for cycles zero through three, between 400 and 500 S_1_ families of WQS were initially screened for general agronomic suitability in a high-plant-density replicated trial in South Central Wisconsin. The same S_1_ families were simultaneously self-pollinated in the breeding nursery. Approximately 50% to 67% of the S_1_ families were discarded based on the stress trial. During the following season, S_1:2_ families descended from random plants within selected S_1_ families are crossed to testers belonging to the Stiff Stalk heterotic group. Resulting topcross hybrids are evaluated at two locations the following summer. Evaluations used standard field plot techniques for silage hybrids to estimate forage yield and composition (Frey *et al.* 2004; Gustafson *et al.* 2010).

Details of the forage composition analysis are provided below. Advancing WQS from cycle four to cycle five involved the same procedures except 200 S_1_ families were initially screened instead of 400–500.

After the fall harvest, the top 20 S_1:2_ families presenting the highest milk production index based on the MILK2006 prediction were selected. These S_1:2_ families were recombined using the bulk entry method, whereby each selected progeny is crossed with each other selected progeny and each cross contributes equally to the next cycle of WQS. In this setup, population improvement and inbred development occurred simultaneously as superior finished (S_6_) lines were identified through the process of selfing, topcrossing, and evaluating.

### Phenotypic data

Starting in 1997, WQS silage yield trials were conducted to select the best 20 S_2_ families for advancing the WQS to the next cycle. WQS C0, C1, C2, C3, and C4 were trialed in 1997, 2000, 2003, 2006, and 2010–2011, respectively. All trials were planted at either West Madison Agricultural Research Station (WMARS; Madison, WI) or Arlington Agricultural Research Station (AARS; Arlington, WI) or both. In 1997, a trial of WQS C0 S_2_ topcross families was planted at one location, WMARS, with four replications. In 2000, a trial of WQS C1 S_2_ topcross families was planted at two locations, WMARS and AARS, with two replications per location. The WQS C2 and C3 S_2_ topcross families were evaluated at WMARS and AARS using three replications at each location in 2003 and 2006, respectively. However, the AARS location in 2006 was abandoned because of a severe windstorm that caused extensive lodging. In 2010, a trial of WQS C4 S_2_ topcross families was planted at WMARS and AARS with two replications, but data quality from AARS in 2010 was very poor and it was therefore discarded. The WQS C4 trial was replanted at WMARS in 2011 using two replications to provide an additional environment for evaluation. All trials consisted of two-row plots, 6.08 m long and with 0.76 m spacing between rows, arranged in a randomized complete block design. Planting densities were common for silage production in the region. Different testers were used across cycles. Tester LH119 was used in WQS C0, LH198 was used in WQS C1, HC33 was used in WQS C2, and LH244 was used in WQS C3 and C4. All testers used are highly related to B73.

Most recently, nutritional quality is evaluated using MILK2006, a summative equation for calculating milk yield based on factors that affect whole-plant maize silage feed quality, including yield, dry matter (DM) content, neutral detergent fiber (NDF) content, NDF digestibility (NDFD), protein, and starch (Schwab *et al.* 2003). Previous versions of this summative equation were utilized in earlier cycles of the WQS selection program. In MILK2006, as well as previous versions, each component (NDF, protein, and starch) is weighted to take into account its respective digestibility. Starch and protein digestibility are traditionally treated as constant, whereas the digestibility of the cell wall, or NDFD, is measured separately for each resulting hybrid. *In vitro* true digestibility (IVTD), acid detergent fiber (ADF), NDF, crude protein, and starch are predicted using a global near-infrared reflectance calibration developed in-house at the University of Wisconsin (https://cornbreeding.wisc.edu/nirs). Wet chemistry procedures to develop the calibration set are described elsewhere (Frey *et al.* 2004; Gustafson *et al.* 2010). The summative equation is then used to develop predictions of milk yield described as kg milk yield Mg^−1^ DM and kg milk yield ha^−1^.

### Genotyping

Remnant seed of available S_2_ families from WQS C0 to WQS C4 and of S_1_ families from WQS C5 were germinated. Immature leaf tissue was collected from 10 individual plants and pooled to represent each of the S_2_ (for WQS C0 to WQS C4) and S_1_ (in the case of WQS C5) families, respectively, selected at each cycle. Genomic DNA was extracted from each sample using a modified CTAB method (Saghai-Maroof *et al.* 1984). Samples were then genotyped using the Illumina MaizeSNP50 BeadChip, an Infinium HD assay (Illumina, Inc. San Diego, CA) with 56,110 SNP markers distributed across the maize genome (Ganal *et al.* 2011). Alleles for each sample were called using the Genotyping Module within the Illumina Genome-Studio software. The built in GenCall data analysis software, which relies on the GenTrain clustering algorithm, was used for automatic clustering and calling of genotypes (Oliphant *et al.* 2002; Fan *et al.* 2003). To maintain only the highest-quality SNPs, a GenCall threshold of 0.6 was used. This filtering resulted in a dataset of 17,719 high-quality SNPs to be used for further analysis. The mean frequency of missing data was 0.07, with a range of 0 to 0.20. Of these markers, 15,646 were polymorphic, with polymorphic markers being defined as those with minor allele frequencies greater than 0.025. Missing marker scores were imputed using Beagle (Browning and Browning 2009) implemented in the R package Synbreed (Wimmer *et al.* 2012). Imputation accuracy was defined as the mean posterior probability of the most likely genotypes and calculated using the *gprobsmetrics* utility in the Beagle package. The average imputation accuracy in this SNP dataset was greater than 99% for all chromosomes.

### Analysis of phenotypic data

Data from different cycles were kept separate and the initial phenotypic data analysis was performed for each cycle separately. The following mixed linear model was fit to the phenotypic data

$$y_{ijk}=\mu +g_{i}+l_{j}+gl_{ij}+b_{k}{}_{\left(j\right)}+\varepsilon_{ijk}$$

where $y_{ijk}$ is the observation of the *i*^th^ family evaluated in the *j*^th^ environment in the *k*^th^ replication; μ is the intercept; *g*_i_ is the effect of the *i*^th^ family; *l*_j_ is the effect of the *j*^th^ environment; *gl*_ij_ is the interaction between the *i*^th^ family and *j*^th^ environment; *b_k_*_(_*_j_*_)_ is the effect of the *k*^th^ replicate nested within the *j*^th^ environment; and $\varepsilon_{ijk}$ is the residual. Environment and replicate effects were modeled as fixed effects. Family and family-by-environment interaction effects were modeled as random effects assumed to be independent and identically distributed. Variance components were estimated using restricted maximum likelihood and best linear unbiased predictions (BLUPs) for each trait were calculated for families. Each cycle was analyzed separately. All calculations were performed using the statistical analysis software ASReml-R (Butler *et al.* 2009).

Variance components were used to calculate broad-sense heritability (*H*) on a family-mean basis as H=σG2σG2+σGE2e+σε2re, where $\sigma_{G}^{2}$ is the variance among families, $\sigma_{GE}^{2}$ is the variance due to family-by-environment interaction effects, $\sigma_{\varepsilon }^{2}$ is the residual variance, *e* is the number of environments, and *r* is the number of replications in each environment.

### Genomic heritability

The proportion of variation among S_2_ family BLUPs across cycles explained by the genomic relationship matrix was calculated. The genomic relationship matrix among all families was calculated as:

$$G=\frac{W_{C}W_{C}’}{2\sum_{l}p_{l}q_{l}}$$

where **W_C_** is the centered genotype matrix, and *p_l_* and *q_l_* are allele frequencies at the *l*^th^ locus (Endelman and Jannink 2012). The following G-BLUP model was fit to the data:

$$\hat{g}=Xb+Zu+e$$

where $\hat{g}$ is the vector of family BLUPs; **b** is a vector of fixed year effects (corresponding to selection cycle); **u** is a vector of random additive genetic values where $u\sim MVN(0,G\sigma_{u}^{2})$; **e** is a vector of residuals; and **X** and **Z** are incidence matrices relating **b** and **u** to $\hat{g}$, respectively. All calculations were made using ASReml-R (Butler *et al.* 2009) and the variance components $\sigma_{u}^{2}$ and $\sigma_{e}^{2}$ were estimated. Genomic heritability was calculated as $h_{G}^{2}=\frac{\sigma_{u}^{2}}{\sigma_{u}^{2}+\sigma_{e}^{2}}$ (De Los Campos *et al.* 2013).

### Association mapping

A genome-wide association analysis for each trait was performed using the model:

$$\hat{g}=Xb+Wm+Zu+e$$

where $\hat{g}$, **X**, **b**, **Z**, **u**, and **e** are as above; **m** is a vector of marker effects; and **W** is a matrix comprising marker scores. The association analysis was implemented using EMMA (Kang *et al.* 2008). A statistical threshold of *P* = 10^−4^ was used to declare significant marker-trait associations. Because a preliminary analysis indicated no markers surpassed this threshold, no further effort was made to better define the statistical threshold to correct for multiple testing.

### Selection mapping and gene diversity

Allele frequencies in WQS C2 and WQS C5 were calculated based on their maximum likelihood estimate, *i.e.*, the observed number of copies of the minor allele divided by twice the number of individuals with an observed genotype. WQS C2 was utilized rather than WQS C0 or C1 because samples from the earlier cycles of selection did not include enough individuals for reliable estimates of allele frequencies. SNP-specific *F_ST_* values based on a comparison of C2 and C5 were computed according to $\mathrm{Fst}={\text{s}}^{2}/\left(\bar{\text{p}}(1-\bar{\text{p}})+{\text{s}}^{2}/\text{r}\right),$ where *s*^2^ is the sample variance of allele frequency between populations, $\bar{\text{p}}$ is the mean allele frequency across populations, and *r* is the number of populations (Weir and Cockerham 1984).

Significance thresholds were determined via drift simulations of the demographic history of the WQS population, assuming linkage equilibrium between markers. Simulations were conducted within R (R Core Team 2014). For each SNP in C2, a simulated allele frequency in C5 was created according to the WQS selection protocol, incorporating generations of selfing, crossing, evaluating, and recombining based on the precise number of individuals utilized at each step in the WQS program. Allele frequencies in C2 and C5 were used to calculate simulated *F_ST_* values for each SNP. The maximum *F_ST_* value observed across SNPs was recorded. This process was repeated 1000 times. The 95% quantile of maximum *F_ST_* values observed from simulations was taken as a simulated 95% significance threshold that accounts for multiple testing across all 17,590 SNPs. The R script used for simulations is provided (Supporting Information, File S1).

The above simulations assumed linkage equilibrium. This is a conservative approach because it allows for more independent tests than may truly be appropriate; since SNPs are inherited in linked segments, the true number of independent loci may be lower than the number of SNPs. To explore this possibility, the effective number of markers (*M_eff_*), were computed with the *simpleM* software (Gao *et al.* 2008). The above simulation strategy was again used, but with the results of *simpleM* incorporated. To achieve this, the C2 starting population was simulated by sampling *M_eff_* SNPs, where *M_eff_* was obtained utilizing the parameters PCA_cutoff = 0.99 and 0.95. Ultimately, utilizing the *M_eff_* SNPs instead of the total number of SNPs did not result in a substantive difference in the estimated significance threshold. Therefore, thresholds obtained via linkage equilibrium simulations were utilized throughout this experiment.

We also performed an enrichment analysis to assess if there is an excess of loci displaying a large change in allele frequency. This was achieved by using the previously described simulations to identify the expected 95% and 99% quantiles of *F_ST_* over the course of the experiment. Then, the observed proportion of loci exceeding these quantiles was computed. Theoretically, 5% and 1% of loci will exceed these quantiles, assuming no selection.

Gene diversity (*D*; *i.e.*, expected heterozygosity) was estimated for each SNP and for each selection cycle from WQS C2 through C5 using ${\hat{D}}_{lc}=1-({\hat{p}}_{lc}^{2}+{\hat{q}}_{lc}^{2})$_,_ where ${\hat{D}}_{lc}$ is the gene diversity estimate for the *l*^th^ SNP in the *c*^th^ selection cycle, ${\hat{p}}_{lc}^{}$ is the allele frequency of that SNP, and ${\hat{q}}_{lc}=1-{\hat{p}}_{lc}$ (Weir 1996).

## Results

### Association mapping

A total of 648 individuals from the WQS population were genotyped for this study. Most individuals came from WQS C2 to C5, whereas only 16 individuals were genotyped from WQS C0 and C1 (Table 1) because of germination problems most likely a result of seed source age. Both genotype and phenotype data were available for between 240 and 300 families from WQS C1 to C4, depending on the trait.

**Table 1:** Individuals with genotypic and phenotypic data included in the evaluation of the WQS population

	Both Phenotypic and Genotypic Data							
Cycle	Silage Yield	Dry Matter	NDF*^a^*	ADF*^a^*	IVTD*^a^*	CP*^a^*	Starch	Genotypic Data
WQS C0	0	0	0	0	0	0	0	5
WQS C1	6	6	6	0	6	6	6	11
WQS C2	0*^b^*	60	60	60	60	60	60	163
WQS C3	80	80	79	79	79	79	79	88
WQS C4	154	154	114	114	114	114	114	170
WQS C5	0	0	0	0	0	0	0	211
Total	240	300	259	253	259	259	259	648

Population was selected for five cycles for silage yield and compositional traits.

a NDF, neutral detergent fiber; ADF, acid detergent fiber; IVTD, in vitro true digestibility; CP, crude protein.

b Data for silage yield from WQS C2 was not included because of zero heritability (see Table 2).

Trait means and ranges are displayed in Table 2 to provide an overview of the phenotypic data analyzed for the association analysis. The range in silage yield within a given cycle was, on average, 45% of the mean. On the other extreme, the range in IVTD within a given cycle was, on average, 8.5% of the mean. Broad-sense heritabilities on a family-mean basis for each cycle were mostly moderate to high (Table 3). An exception was the *H* for yield in WQS C2. Broad-sense heritability was generally higher for dry matter and starch, and lower for ADF and NDF. The $h_{G}^{2}$ within cycles was mostly low except for dry matter, CP in WQS C2, and starch WQS C4. The $h_{G}^{2}$ across cycles was also low. This indicates that while the genotype accounts for a fair proportion of the phenotypic variation within a given cycle or year of evaluation, little of the variation is captured by an additive relationship matrix.

**Table 2:** Mean, minimum, and maximum of each trait in each year (cycle) of evaluation

		WQS C1				WQS C2				WQS C3				WQS C4			
Trait	Units	Mean	Min	Max	S/σ_P_*^a^*	Mean	Min	Max	S/σ_P_	Mean	Min	Max	S/σ_P_	Mean	Min	Max	S/σ_P_
Silage yield	Mg ha^−1^	7.2	5.7	8.5	0.74	8.9	8.5	10.5	0.24	9.7	5.3	12	1.17	8.7	6.1	10.5	0.81
Dry matter	%	32.1	26.6	42.0	0.07	39.5	33.5	49.2	−0.34	34.1	29.3	39.8	0.44	36.9	30.1	43.7	0.31
NDF	%	53.3	48.2	58.5	−0.23	50.5	46.7	55.4	−1.11	46.9	42.9	51.1	−0.35	44.8	42.0	49.3	0.23
ADF	%	—*^b^*	—	—	—	26.2	23.6	29.7	−1.04	24.7	22.1	27.7	−0.42	23.0	21.4	25.8	0.24
IVTD	%	70.9	65.9	75.0	0.26	82.4	80.0	86.2	1.07	78.6	76.1	81.5	0.26	81.1	78.2	83.7	−0.33
CP	%	7.2	6.3	8.0	−0.18	8.0	7.2	8.8	0.15	7.0	6.2	8.1	−0.24	6.9	6.2	7.5	0.51
Starch	%	21.9	12.7	30.6	−0.01	27.9	20.8	33.7	0.61	30.5	20	34.9	0.37	31.4	25.2	35.7	0.04

a A standardized selection differential was calculated for each cycle by dividing the selection differential by the phenotypic SD.

b ADF was not measured in WQS C1.

**Table 3:** Broad-sense heritability on a family-mean basis (*H*) and genomic heritability ($h_{G}^{2}$) for each trait in each selection cycle of the WQS selection program and $h_{G}^{2}$ across cycles

	Silage Yield		Dry Matter		NDF		ADF		IVTD		CP		Starch	
Cycle	*H*	$h_{G}^{2}$	*H*	$h_{G}^{2}$	*H*	$h_{G}^{2}$	*H*	$h_{G}^{2}$	*H*	$h_{G}^{2}$	*H*	$h_{G}^{2}$	*H*	$h_{G}^{2}$
WQS C1	0.43	—*^a^*	0.73	—	0.33	—	—*^b^*	—	0.38	—	0.54	—	0.58	—
WQS C2	0	0	0.71	0.53	0.52	0.04	0.49	0.05	0.52	0.01	0.73	0.57	0.71	0.21
WQS C3	0.59	0.16	0.82	0.42	0.34	0	0.35	0	0.48	0	0.60	0.10	0.66	0.31
WQS C4	0.33	0.06	0.64	0.69	0.41	0	0.41	0	0.59	0.27	0.32	0	0.61	0.81
Across cycles	—	0.11	—	0.42	—	0.01	—	0.06	—	0.02	—	0.23	—	0.18

a Not enough individuals were genotyped in cycle 1 to calculate $h_{G}^{2}$.

b ADF was not measured in WQS C1.

Using phenotypic data from historical field trials, an association analysis was performed to identify important genomic regions controlling variation for these yield and compositional traits. Unfortunately, no statistically significant associations (*P* < 10^−4^) were made for any trait (Figure S1).

### Selection mapping

A wide range of *F_ST_* values was observed between SNPs. Since the selection protocols and number of selected individuals at each generation were recorded throughout this experiment, this enabled a simulation-based approach for identifying significance thresholds for the boundaries of *F_ST_* expected to result from drift alone. The 20 S_2_ families selected within each cycle led to a strong bottleneck that the population repeatedly experienced, resulting in a high significance threshold. To obtain 95% and 99% probabilities of no false positives, respectively, significance thresholds were set at *F_ST_* = 0.743 and 0.707.

**Figure 2:**
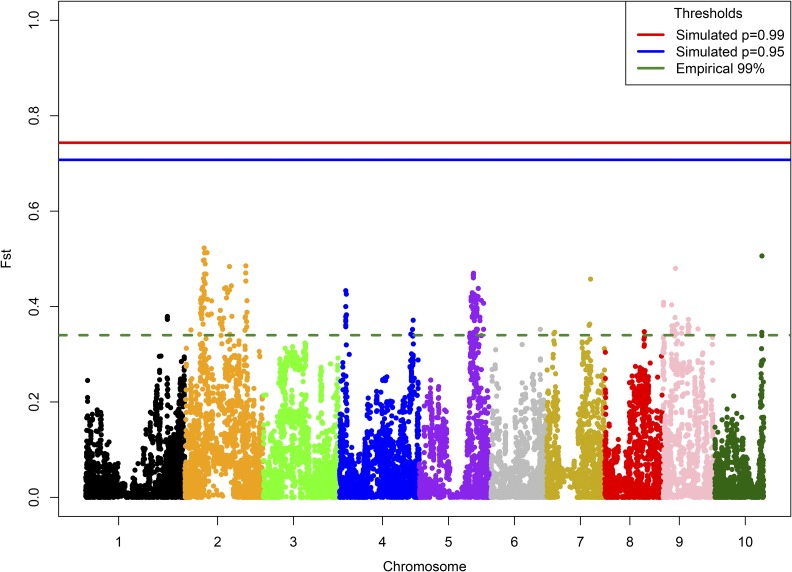
*F_ST_* values between WQS cycle 2 and WQS cycle 5, computed for each SNP. The dashed green line depicts an empirical 99% outlier threshold. Blue and red lines show simulation-based multiple testing corrected significance thresholds, which control for the magnitude of drift that could reasonably be expected according to the selection protocol that was used.

There were no SNPs that exceeded these significance thresholds (Figure 2). Often, much more lenient outlier thresholds are utilized for selection-mapping experiments. Outlier thresholds involve comparing the observed data with its own empirical distribution, thereby guaranteeing that a specified proportion of the data is identified as candidates for selection. Utilizing a 99% outlier threshold in this study would have meant setting the significance threshold at *F_ST_* = 0.340 and identifying 175 “significant” SNPs. Simulations demonstrated that this significance value is substantially lower than the effects of drift may allow.

Additionally, by evaluating drift simulations without accounting for multiple testing, we determined that it is expected that 5% and 1% of SNPs will exceed *F_ST_* values of 0.214 and 0.328, respectively, due to drift alone. We used these values to assess whether there is enrichment for high-*F_ST_* SNPs in the data. We observed that 6.139% and 1.137% of SNPs exceed these uncorrected thresholds, respectively, indicating there is little evidence of enrichment for SNPs displaying high *F_ST_*.

### Reduction of gene diversity

**Figure 3:**
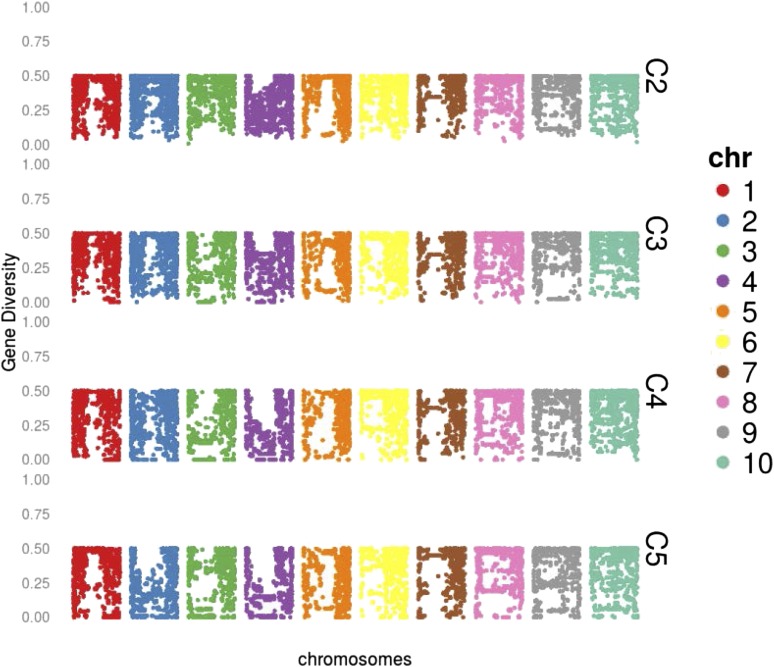
Gene diversity for each SNP evaluated in the Wisconsin Quality Synthetic selection program from cycle 2 (WQS C2) to C5

Despite no strong signatures of selection and marker-trait associations, an examination of *D* for each locus shows that reductions were not uniform across the genome (Figure 3). A large reduction in *D* was observed in regions on chromosomes 2 (∼132 million bp), 3 (∼55 million bp), and 4 (∼78 million bp). These regions of relatively greater loss in diversity were defined visually by examining the *D* plots in Figure 3. Average *D* across all loci was reduced from 0.352 in C2 to 0.285 in C5. Although average genome-wide *D* was only reduced by 19% from C2 to C5, average *D* in these regions on chromosomes 2, 3, and 4 was reduced by 62%, 79%, and 67%, respectively. The large region on chromosome 2, for example, had an average *D* of 0.355 in C2, which is very close to the average genome-wide *D* in C2. By C5, however, the average *D* was only 0.135, which is well below 1 SD of *D* (genome-wide SD = 0.131).

## Discussion

The first objective of this study was to identify loci controlling variation for traits important to silage breeding using a combination of association and selection mapping. Despite moderate to high entry-mean heritabilities within cycles (Table 2) and documented genetic gain for silage yield and composition in WQS (Frey *et al.* 2004; Gustafson *et al.* 2010), no significant results were obtained.

The genetic complexity underlying silage quality and yield is antagonistic to identifying loci contributing to variation and therefore selection response. Although the genetics underlying mechanisms involved in cell wall digestibility could be complex, the dependence of silage quality on grain content, which is related to grain yield, surely makes silage quality increasingly complex. Grain is highly digestible and accounts for approximately 50% of total dry matter of silage (Coors and Lauer 2001). Also, variation in the effectiveness of the ear as a sink can influence stover composition through its effect on dry matter partitioning and transport of sugars to the ear (Coors *et al.* 1997). Stage of plant development at which plants are harvested contributes to variation in silage quality (Jung and Casler 2006). If genetic variation for time to maturity exists within a population, then this variation will be confounded with variation for stover quality. Finally, plant components vary for digestibility and fiber concentrations, and genetic variation exists for digestibility of specific plant components (Hansey *et al.* 2010). Therefore, the genetic complexity of silage quality on a whole-plant basis could easily equal that of grain yield given its dependence on grain yield and plant morphology.

Because starch content in silage and sink-source dynamics are important contributors to quality, and genotype-by-environment (G×E) interactions are an important source of variation for grain yield, it is not surprising that silage compositional traits are highly influenced by G×E interactions, which has been observed in previous studies (Argillier *et al.* 2000; Mechin *et al.* 2001). This source of variation reduces the contribution of the genetic signal to the total variation, decreasing power to detect marker-trait associations and selection signatures across years. On top of possible strong G×E effects, epistatic interactions could reduce the contribution of main allelic effects, and thus result in a loss of power for making associations. Although comparing variance components and thus heritabilities is fraught with issues because of high standard errors, examination of Table 3 shows that the proportion of variation accounted for by the additive genomic relationship matrix is low relative to the entry-mean broad-sense heritability in most cases. This suggests the importance of interactions underlying the variability for these traits, both epistatic interactions within cycles as well as G×E interactions across cycles. Another confounded source of variation is allele-by-tester interactions. As noted in *Materials and Methods*, different testers were used in the different cycles, opening the possibility for tester interaction to dilute the main allelic effects. The testers used were all highly related, being B73 types, and therefore the importance of this source of variation is likely less than if unrelated testers were used.

We demonstrate that although significant genetic gain has been realized for important silage traits within WQS, no strong selection signature was left on the genome. There are at least two reasons for this finding. First, the genetic signal underlying variation for silage yield and composition is highly complex, likely comprising many small main and interaction effects distributed across the entire genome. This hypothesis is supported by the lack of marker-trait associations found in this study. Second, it is possible that selection acted on standing genetic variation caused by old mutations, meaning that a casual polymorphism is not necessarily associated with any particular haplotype. Such soft selective sweeps (Hermisson and Pennings 2005) do not leave a strong selection signature and are difficult to detect using molecular markers.

The lack of a strong selection signature found by this study is in good company among other similar findings on complex traits in agricultural species. Kemper *et al.* (2014) found little to no signature on the genome of cattle left by selection for milk yield, despite enormous genetic gain for this trait, and large differences between cattle breeds. Likewise, selection for grain yield in maize has left only very subtle, if any, selection signatures (Gerke *et al.*, in press; Van Heerwaarden *et al.* 2012). Once again, this is despite substantial genetic gain for grain yield accomplished within both a recurrent selection program (Gerke *et al.*, in press) and commercial breeding (Van Heerwaarden *et al.* 2012).

Given that genetic gain has occurred (Frey *et al.* 2004; Gustafson *et al.* 2010), these observations indicate that the gain realized has been accomplished through subtle allele frequency shifts at many loci. It is encouraging to know that breeders are able to simultaneously increase the frequency of many small-effect alleles, therefore achieving genetic gain on highly complex traits. However, great difficulty in figuring out the causal mechanisms underlying genetic gain for complex traits limits our understanding of the genetics underlying selection response. It is clear that new and more powerful methods are required to identify signatures left by selection on highly polygenic traits. Researchers in population genomics have realized this and have begun developing such methods (Berg and Coop 2014).

Another implication of this study is that caution should be taken when using historical phenotypic data from recurrent selection programs for association mapping of complex traits. While we recognized our power was limited because of only modest population sizes (Table 1), we believed, based on the moderate to high *H*, the trait data from individual cycles was of high-enough quality to detect marker-trait associations. Clearly, that was a wrong assumption. Little of the phenotypic variance across cycles (and thus years) was additive genetic variance, with the majority likely being caused by genetic-by-year interactions given the complexity of the silage compositional traits and their interaction with grain yield. Our experience suggests that historical data are of limited value for association genetics on complex traits prone to genotype-by-year interactions. We recommend that all genotypes be re-evaluated across multiple years and locations to maximize power for detecting associations. It is recognized that the dataset size used herein is relatively small compared with some other historical datasets, and historical data could be useful if vast quantities are available (Vaughn *et al.* 2014).

Recurrent selection is a systematic method to increase allele frequency of a base population, and therefore increases the probability a superior inbred line is derived from that base population through selection and inbreeding (Hallauer 1990). Recurrent selection in plant breeding generally involves the selection of multiple individuals or families (typically 10–50) for recombination each cycle. A theoretical advantage of recurrent selection compared with simple inbreeding and selection is that genetic variation is maintained, leading to sustained genetic gain over time (Bernardo 2010). Response to recurrent selection has reportedly continued after many cycles (Dudley and Lambert 2004; Keeratinijakal and Lamkey 1993; De Leon and Coors 2002). Using molecular markers, several studies on maize populations undergoing recurrent selection have shown that observed average losses in gene diversity (*i.e.*, expected heterozygosity) are approximately equal to that expected by theory assuming genetic drift and a given effective population size (Lamkey and Lorenz 2014; Labate *et al.* 1999; Hinze *et al.* 2005; Butruille *et al.* 2004; Romay *et al.* 2012). None of the aforementioned studies, however, used marker densities great enough to observe variation in diversity loss across the genome. By genotyping individuals from multiple cycles of selection of the WQS with more than 15,000 high-quality, informative SNPs, we were able to assess the degree to which gene diversity reductions vary across the genome. Very few studies in maize have examined the effects of recurrent selection using high-density SNPs (Gerke *et al.*, in press; Beissinger *et al.* 2014; Hirsch *et al.* 2014). Although we observed that most loci followed expectations, a few genomic regions experienced substantial loss of diversity presumably through the combination of chance and the low number of crossovers occurring on each maize chromosome. A similar observation was made by Gerke *et al.*, in press. Using the same Illumina Infinium array, these authors observed that a number of large genomic regions within the BSSS/BSCB1 recurrent selection populations became completely fixed for one haplotype after 16 cycles of selection. Based on the selection procedures used, it was difficult to determine if this was caused by drift or selection. It appears that the regions on chromosomes 2, 3, and 4 are headed for the same fate in the WQS recurrent selection program.

Given the erratic nature of drift in recurrent selection programs with relatively small effective population sizes, combined with the limited number of crossovers occurring on any given maize chromosome each generation, it is entirely possible for a population to become fixed for one haplotype across a large swath of genomic space. This means that while genome-wide diversity in a population may be seemingly satisfactory for continued progress, diversity within specific regions could be inadequate. If these regions harbor loci important for traits of interest, then genetic gain would be compromised and the population would be prevented from reaching its full potential. A major advantage to the routine use of high-density markers in a breeding program would be the ability to monitor genomic variability in allelic diversity and, ultimately, to identify any regions that would benefit from targeted injections of allelic diversity.

## Conclusions

This is the first report of an analysis on genetic gain for silage yield and composition at the genomic level. No strong genomic signatures were left by selection on silage yield and quality in the WQS, likely due to the complexity underlying these traits. The role of selection on standing genetic variation could also be contributing to the lack of strong signatures. Variation in loss of diversity through drift was observed across the genome. A few large regions experienced much greater loss in diversity than what is expected, indicating limited recombination and population sizes in recurrent selection programs could lead to fixation of large swaths of the genome.

## 

## Supplementary Material

Supporting Information
