# Supplementary material for: Selection for Silage Yield and Composition Did Not Affect Genomic Diversity Within the Wisconsin Quality Synthetic Maize Population
Source: G3 (Bethesda). 2015 Feb 2;5(4):541–9. doi: 10.1534/g3.114.015263 (PMC4390570; doi:10.1534/g3.114.015263)
Supplement: Supporting Information [file supp_5_4_541__index.html]

Selection for Silage Yield and Composition Did Not Affect Genomic Diversity Within the Wisconsin Quality Synthetic Maize Population — Supporting Information 

# Selection for Silage Yield and Composition Did Not Affect Genomic Diversity Within the Wisconsin Quality Synthetic Maize Population

## Supporting Information for Lorenz *et al.*, 2015

**Files in this Data Supplement:**

- Figure S1 - Manhattan plots produced from an association analysis on silage compositional traits neutral detergent fiber (NDF), crude protein (CP), *in vitro* true digestibility (IVTD), and starch. (PDF, 643 KB)
- File S1 - R codes (.R, 6 KB)
- File S2 - WQS GWAS and Selection Mapping Supplemental Data (.zip, 3 MB)
