# Supplementary material for: Selection for Silage Yield and Composition Did Not Affect Genomic Diversity Within the Wisconsin Quality Synthetic Maize Population
Source: G3 (Bethesda). 2015 Feb 2;5(4):541–9. doi: 10.1534/g3.114.015263 (PMC4390570; doi:10.1534/g3.114.015263)
Supplement: Supporting Information [file supp_g3.114.015263_FigureS1.pdf]

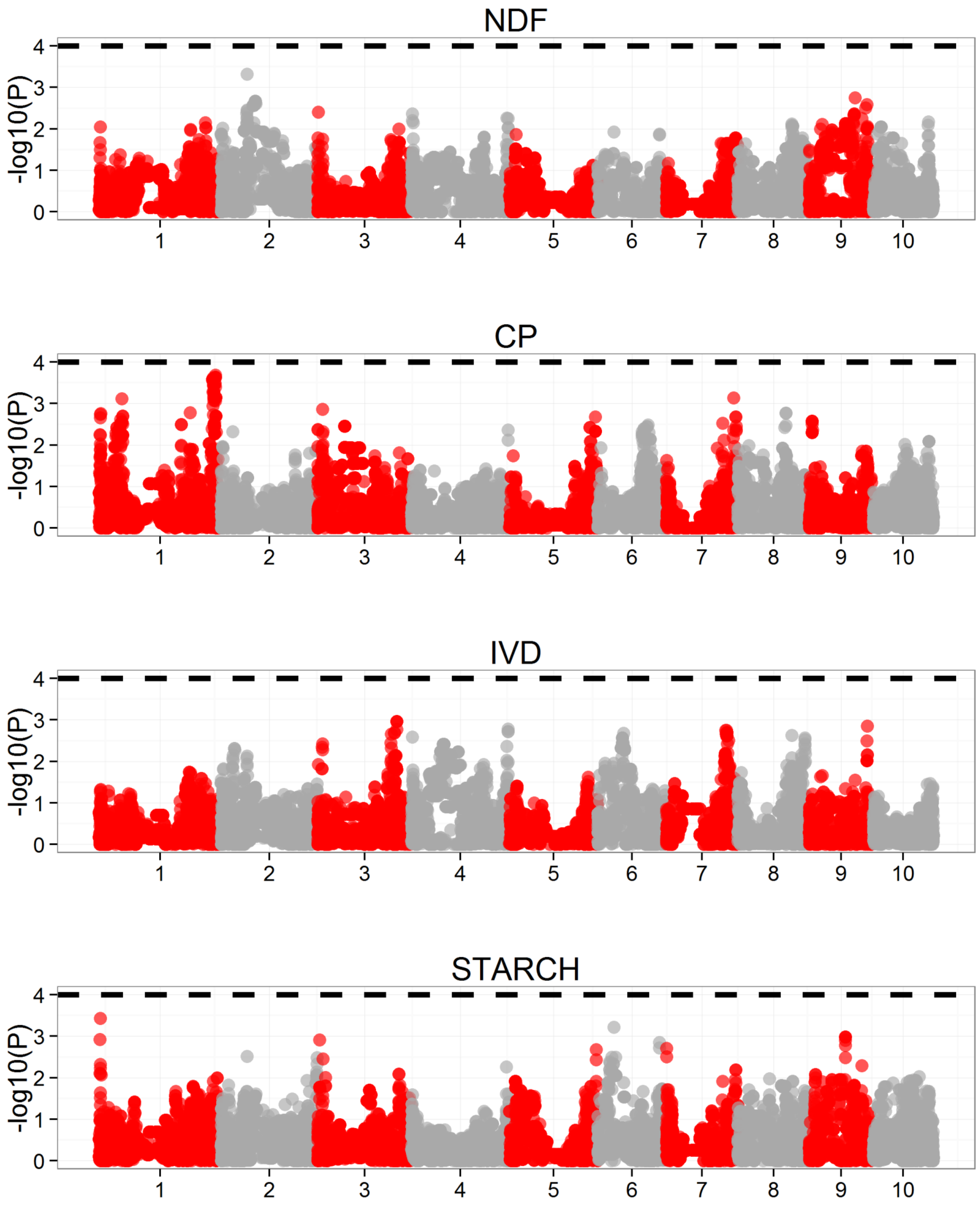
NDF
CP
IVD
STARCH
-log10(P)
0
1
2
3
4
1
2
3
4
5
6
7
8
9
10

Figure S1 Manhattan plots produced from an association analysis on silage compositional traits neutral detergent fiber (NDF), crude protein (CP), *in vitro* true digestibility (IVTD), and starch. Numbers along the *x*-axis represent the 10 chromosomes of maize. Dashed lines are placed at the arbitrary statistical threshold of $P = 10^{-4}$.
